# β-Catenin Is Required for Prostate Development and Cooperates with *Pten* Loss to Drive Invasive Carcinoma

**DOI:** 10.1371/journal.pgen.1003180

**Published:** 2013-01-03

**Authors:** Jeffrey C. Francis, Martin K. Thomsen, Makoto M. Taketo, Amanda Swain

**Affiliations:** 1 Division of Cancer Biology, Institute of Cancer Research, London, United Kingdom; 2 Department of Pharmacology, Graduate School of Medicine, Kyoto University, Kyoto, Japan; Fred Hutchinson Cancer Research Center, United States of America

## Abstract

Prostate cancer is a major cause of male death in the Western world, but few frequent genetic alterations that drive prostate cancer initiation and progression have been identified. β-Catenin is essential for many developmental processes and has been implicated in tumorigenesis in many tissues, including prostate cancer. However, expression studies on human prostate cancer samples are unclear on the role this protein plays in this disease. We have used *in vivo* genetic studies in the embryo and adult to extend our understanding of the role of β-Catenin in the normal and neoplastic prostate. Our gene deletion analysis revealed that prostate epithelial β-Catenin is required for embryonic prostate growth and branching but is dispensable in the normal adult organ. During development, β-Catenin controls the number of progenitors in the epithelial buds and regulates a discrete network of genes, including *c-Myc* and *Nkx3.1*. Deletion of β-Catenin in a *Pten* deleted model of castration-resistant prostate cancer demonstrated it is dispensable for disease progression in this setting. Complementary overexpression experiments, through *in vivo* protein stabilization, showed that β-Catenin promotes the formation of squamous epithelia during prostate development, even in the absence of androgens. β-Catenin overexpression in combination with *Pten* loss was able to drive progression to invasive carcinoma together with squamous metaplasia. These studies demonstrate that β-Catenin is essential for prostate development and that an inherent property of high levels of this protein in prostate epithelia is to drive squamous fate differentiation. In addition, they show that β-Catenin overexpression can promote invasive prostate cancer in a clinically relevant model of this disease. These data provide novel information on cancer progression pathways that give rise to lethal prostate disease in humans.

## Author Summary

Prostate cancer is a major cause of male death in the Western world, but few genes involved in this disease have been identified. We have undertaken an in-depth *in vivo* analysis in the prostate of the β-Catenin protein, which has been shown to be important in many processes during embryogenesis and has been implicated in tumorigenesis. Our studies demonstrate that β-Catenin is essential for prostate development but is dispensable in the normal adult organ. Analysis of a mouse model of a frequently mutated human prostate tumour suppressor, *Pten* loss, revealed that β-Catenin is not required for neoplastic formation in this model, even in castrated conditions. However, increased β-Catenin levels can cooperate with *Pten* loss to promote the progression of aggressive invasive prostate cancer together with squamous metaplasia. These data uncover the role of β-Catenin in the prostate and provide new insights on how pathways interact to drive human prostate cancer.

## Introduction

Prostate cancer is the most common male cancer in the developed world, and a leading cause of cancer-related death in men. To date, few common genes that promote prostate cancer progression have been identified, including the tumour suppressor *PTEN* (phosphatase and tensin homolog deleted on chromosome 10), the gene rearrangement *TMPRSS2-ERG*, and the oncogene *C-MYC* [1]. There is a need to determine additional drivers of prostate cancer that could be the targets of new therapies, and to determine the genetic and molecular interaction between these signalling pathways. One approach successfully used to identify and study the function of candidate cancer genes has been to investigate genes during embryonic development of the organ. This is a useful method as many genes and pathways involved in development are also reactivated in cancer initiation and progression [2]. Examples of genes that have been found to be important during both prostate development and tumorigenesis include *Sox9*, *Hoxb13* and *Nkx3.1* [3], [4], [5]

The prostate develops from the endodermally derived urogenital sinus (UGS) in response to androgens that signal through the androgen receptor (AR) [6]. This occurs at embryonic day (E) 16.5–17.5 in the mouse, when the epithelium buds out and grows into the surrounding mesenchyme. This process requires complex epithelial-mesenchymal interactions and involves signalling pathways such as androgens, FGF and SHH [6]. The buds then elongate, branch, canalize and undergo cytodifferentiation to become secretory after birth. The adult prostate epithelium is composed of p63 expressing basal cells, AR positive luminal cells and rare neuroendocrine cells [7]. The adult normal and neoplastic prostate remains responsive to androgens and castration is a first line of therapy for patients with prostate cancer. However, after an initial response to androgen withdrawal, the tumour grows in a castration-resistant phase [8].

The cytoplasmic protein β-Catenin (encoded by the *CTNNB1* gene) is crucial in many steps of embryogenisis and is involved in a number of cancers [9]. β-Catenin forms part of the adherens junction with E-Cadherin and is also a component of canonical WNT signalling [9]. In the absence of WNT ligand, a destruction complex of Axin, APC, GSK3β and CK1α promotes the phosphorylation and subsequent degradation of β-Catenin via the ubiquitin pathway. When WNT ligand binds to the Frizzled/LRP receptor complex, the destruction complex is destabilized and GSK3β is unable to phosphorylate β-Catenin. This leads to an accumulation of β-Catenin that translocates to the nucleus and interacts with the transcription factors TCF/LEF to activate target genes.

Currently, the function of β-Catenin in human prostate cancer is unclear [10]. *CTNNB1* mutations in prostate cancer occur rarely, in only 5% of cases [11]. It has been observed that β-Catenin expression and localization change during human prostate cancer progression, however, results are inconsistent. Several studies have seen an increase in β-Catenin expression and nuclear localization in late stage cancer samples, while others have reported a loss in nuclear expression in advanced tumours [12], [13], [14], [15], [16]. In addition to its role in WNT signalling, β-Catenin can act as a co-factor with AR, suggesting it has a role in castration-resistant disease. In prostate cancer cell lines, β-Catenin enhances androgen-stimulated AR transcriptional activation and increases sensitivity to low levels of androgens and to non-androgen ligands [17], [18], [19], [20], [21], [22]. Nuclear localization of β-Catenin may also result in increased complexes between AR and β-Catenin in prostate cancer cells, changing target gene activation [18], [23]. Furthermore, castration-resistant growth of a prostate cancer xenograft model results in increased β-Catenin and AR expression, co-localization in the nucleus and physical interaction of the proteins [24]. Mouse models have demonstrated that activating β-Catenin, through generation of a non-degradable form of this protein, leads to prostate neoplasia and squamous transdifferentiation [25], [26], [27]. Mouse prostates expressing this stabilized form of β-Catenin in combination with either the SV40 large T-antigen (LPB-Tag), which inactivates p53 and Rb, or with a mutated *K-ras* form invasive carcinoma [28], [29].

*PTEN* is frequently altered in prostate cancer, with mutations and/or deletions found in 30% of primary cancers and 63% of metastatic prostate tumours [30], [31]. PTEN is a phosphatase that negatively regulates the phosphatidylinositol-3-kinase/Akt (PI3K/Akt) pathway [32]. PTEN loss promotes phosphorylation of Akt through PI3K, which in turn phosphorylates multiple targets including GSK3β. Activation of this pathway results in an increase in cell proliferation, cell survival and protein synthesis [32]. Evidence suggests that β-Catenin can interact with the PI3K/Akt pathway following PTEN loss, through the inactivation of GSK3β and stabilization of β-Catenin. *PTEN* null prostate cancer cells have increased nuclear β-Catenin expression, TCF promoter activity and expression of the β-Catenin regulated gene Cyclin D1, which are suppressed upon re-expression of wild type *PTEN* [33], [34].

To better define the function of β-Catenin in the normal and neoplastic prostate we have used *in vivo* gene deletion and activation (through generation of a stabilized form of the protein) in the embryonic and adult mouse. Loss of *Ctnnb1* in epithelia during mouse prostate development results in failure of bud outgrowth and branching, while β-Catenin activation leads to squamous transdifferentiation through an androgen-independent mechanism. Surprisingly, deletion of *Ctnnb1* in the adult prostate had no effect on normal prostate homeostasis. To study its function in prostate cancer we analysed the role of β-Catenin in the context of the frequently deleted *PTEN* gene. We demonstrate that β-Catenin expression is elevated after *PTEN* loss but plays no function in prostate cancer in intact or castrated animals. However, increasing the level of β-Catenin in combination with *PTEN* deletion leads to highly invasive prostate tumours with persistent squamous metaplasia.

## Results

### β-Catenin is required for prostate bud growth

**Figure 1:**
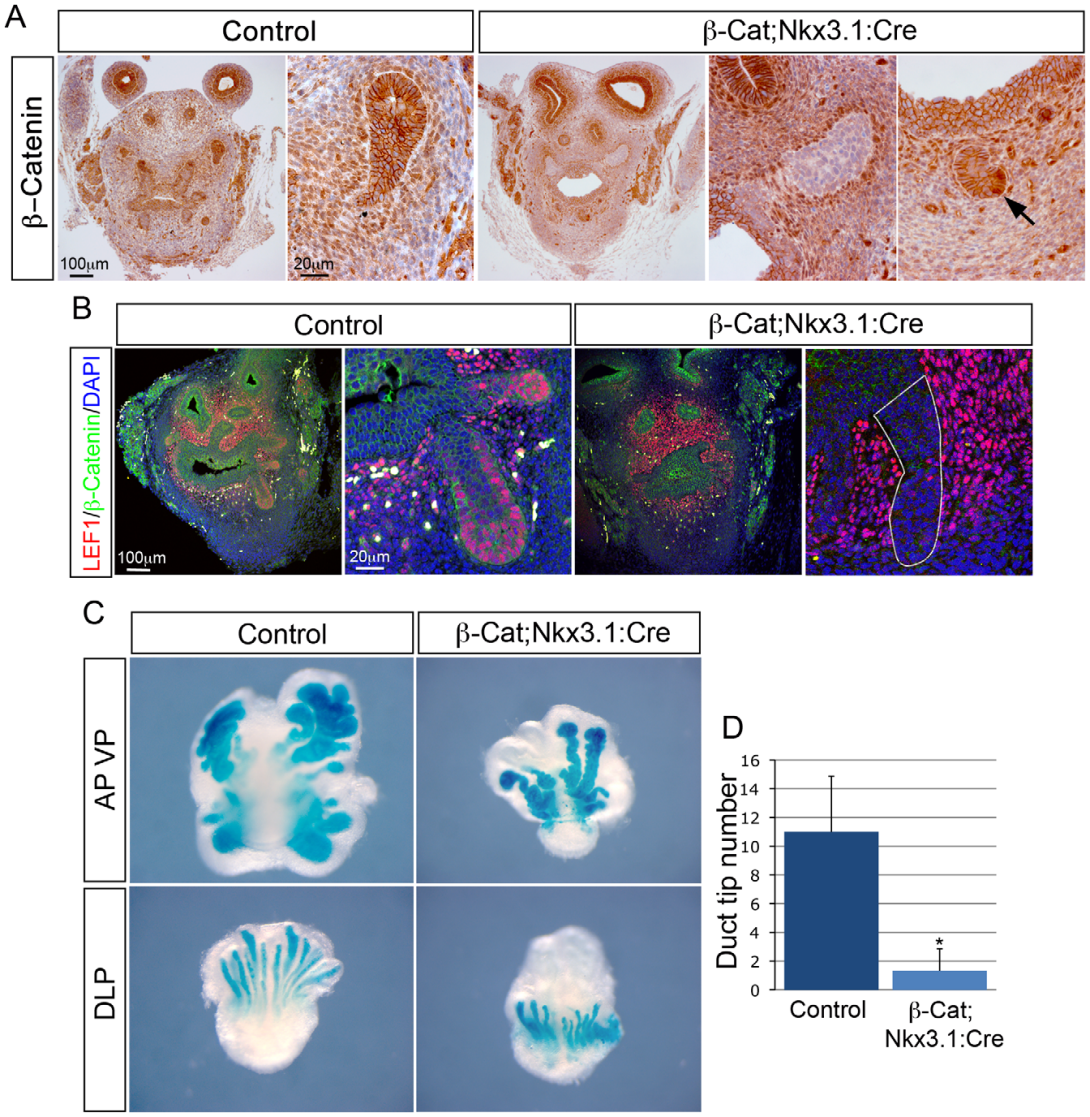
β-Catenin is required for prostate growth and branching. (A) immunohistochemistry (IHC) on sections of *β-Cat;Nkx3.1:Cre* mutant and control E18.5 UGS with a β-Catenin antibody. Adjacent panels show high magnification of buds. Note the strong staining in the buds of control prostates that is lost in most cells of mutant prostates. Far right panel is high magnification of a bud that has not lost β-Catenin, indicated with a black arrow. (B) double IHC for β-Catenin and LEF1 on *β-Cat;Nkx3.1:Cre* and control E18.5 UGS sections. Strong nuclear LEF1 expression is observed in control prostatic buds, but lost in mutant buds. Adjacent panels show buds at high magnification. White outline indicates bud. White cells are autofluorescence from red blood cells. (C) X-gal staining of *ROSA-LacZ β-Cat;Nkx3.1:Cre* and control prostate organ cultures grown for 5 days. (D) quantitative analysis shows a significant decrease in the total number of prostatic ducts in *β-Cat;Nkx3.1:Cre* mutants (p = 0.0068, n = 5). Error bars represent standard deviation.

To determine the function of β-Catenin in the prostate during development, we used a conditional gene deletion approach to specifically delete *Ctnnb1* in prostate epithelia. We used the *Nkx3.1:Cre* mouse line to drive the expression of *Cre* recombinase in epithelial cells at E17.5 just after bud induction, which in combination with a *loxP* containing *Ctnnb1* allele (*β-Cat*) results in the excision of exons 2 to 6 and the production of a non-functional protein [35]. As the *Nkx3.1:Cre* mouse line is a knock-in allele and, therefore, heterozygous for *Nkx3.1*, *β-Cat;Nkx3.1:Cre* heterozygous animals were used as controls. At E18.5, β-Catenin is expressed at the membrane and in the cytoplasm of epithelial cells throughout the buds of control prostates and is lost in most cells of *β-Cat;Nkx3.1:Cre* mutant buds, although a small number of cells retain expression (Figure 1A). At this stage, LEF1, a direct transcriptional target of β-Catenin, has strong nuclear expression at the tips of developing control buds, which is lost from most epithelial cells in *β-Cat;Nkx3.1:Cre* prostates (Figure 1B) [36].

**Figure 2:**
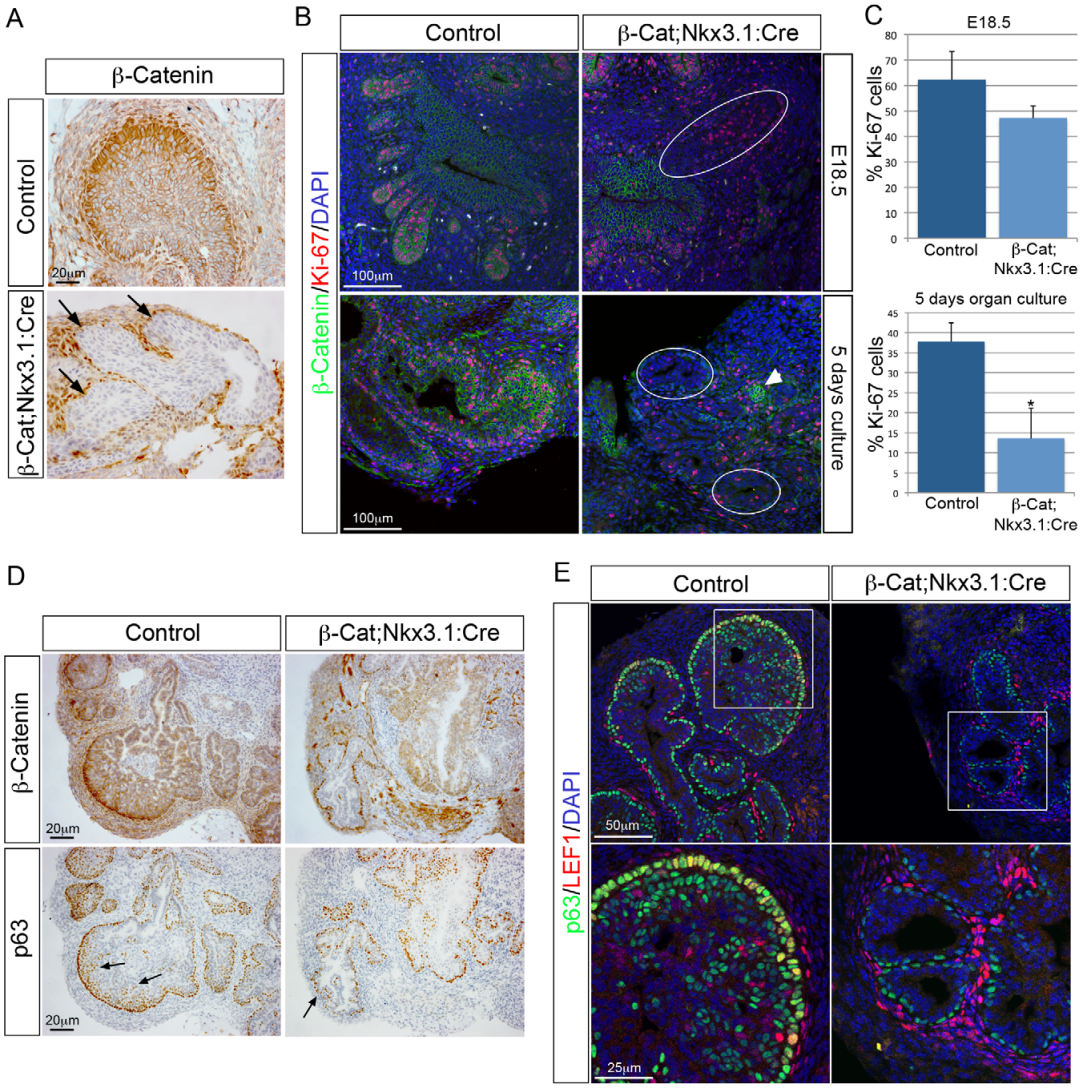
β-Catenin regulates proliferation of prostatic duct progenitors. (A) IHC for β-Catenin on sections of *β-Cat;Nkx3.1:Cre* and control E18.5 UGS that have been grown in organ culture for 5 days. Black arrows indicate buds that have lost β-Catenin expression. (B) double IHC for β-Catenin and Ki-67 on *β-Cat;Nkx3.1:Cre* and control E18.5 UGS sections (top panel) and *β-Cat;Nkx3.1:Cre* and control prostate organ culture sections (bottom panels). White circles indicate mutant buds that have lost β-Catenin. White arrowhead indicates an epithelial bud that has not lost β-Catenin. (C) quantitative analysis of Ki-67 shows there is no significant decrease in proliferation in mutant prostate ducts at E18.5. A significant decrease in proliferation is evident after 5 days in culture, compared to control prostates (E18.5 p = 0.095, 5 days in culture p = 0.0016). (D) β-Catenin and p63 IHC on serial sections of *β-Cat;Nkx3.1:Cre* and control prostate organ cultures. Arrows indicates multiple p63 expressing cells at the tips of control buds that are absent from mutant buds. (E) double IHC for LEF1 and p63 on *β-Cat;Nkx3.1:Cre* and control E18.5 UGS that have been grown in organ culture for 5 days. White boxes indicate position of high magnification images shown below. Error bars represent standard deviation.

All *β-Cat;Nkx3.1:Cre* mutant pups died at birth and showed defects in vertebrae formation, likely a result of *Nkx3.1* expression in paraxial mesoderm [37]. To overcome this, prostates were grown in *ex vivo* organ culture. Prostates were dissected at E18.5, placed on filters and grown such that individual lobes could be assessed. To evaluate epithelial bud development, mutant animals were mated to *ROSA26-LacZ* reporter mice that express LacZ upon Cre mediated recombination [38]. After 5–6 days in culture, control anterior and ventral lobes grew thick buds with multiple branches and dorsal-lateral lobes grew long buds with branches (Figure 1C). In contrast, *β-Cat;Nkx3.1:Cre* anterior lobes grew primary buds that were thinner and lacked branches, while ventral and dorsal-lateral lobes grew small buds with no branches. Quantification of prostate duct tips revealed a significant decrease in *β-Cat;Nkx3.1:Cre* mutants, demonstrating a reduction in branching (Figure 1D). After 5 days of culture, most epithelial cells have lost β-Catenin but a small number of cells still retain protein expression (Figure 2A and 2B). To determine if the smaller mutant prostates are due to a decrease in cellular proliferation the marker Ki-67 was analysed. At E18.5 there is no significant difference in the proliferation of cells in mutant buds (p = 0.095, n = 5) (Figure 2B and 2C). In contrast, after being grown for 5 days in culture, there is a significant decrease in dividing cells in mutant prostates (13%) compared to control prostates (37%) (p = 0.0016, n = 5) (Figure 2B and 2C). At this stage, there is a concomitant decrease in the number p63 positive cells at the tip of *β-Cat;Nkx3.1:Cre* mutant buds (Figure 2D). Control buds have multiple layers of p63 expressing cells at the tip of the bud with many co-expressing LEF1 (Figure 2E). In contrast, *β-Cat;Nkx3.1:Cre* buds have lost epithelial LEF1 expression and a single layer of p63 expressing cells is present. These data show that β-Catenin is required for prostate growth and branching and regulates the number of p63 positive cells at the tip of the buds.

**Figure 3:**
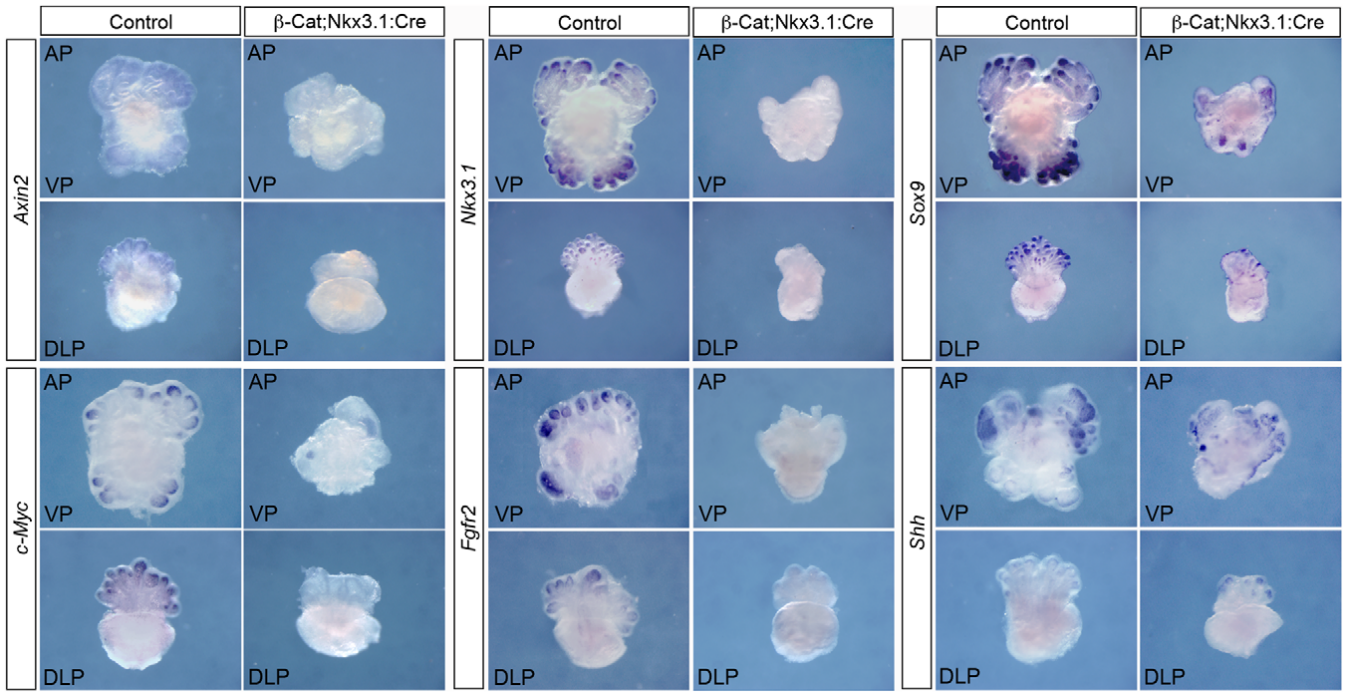
β-Catenin regulates a discrete network of genes during prostate development. WISH analysis of *β-Cat;Nkx3.1:Cre* mutant and control prostate organ cultures grown for 5 days shows expression of *Axin2*, *c-Myc*, *Nkx3.1* and *Fgfr2* are lost in mutant buds, while *Sox9* and *Shh* expression are still present. VP is the ventral lobe, AP is the anterior lobe and DLP is the dorsal lateral lobe.

To identify molecular targets of β-Catenin in prostate bud growth, we analysed the expression of established targets and genes known to be involved in prostate development by whole-mount *in situ* hybridization (WISH) on prostates dissected at E18.5 and grown in organ culture. Two well-described targets of WNT/β-Catenin signalling are *Axin2*, a negative regulator of the pathway, and *c-Myc*, a transcription factor that regulates multiple processes including cell proliferation and differentiation. *Axin2* is expressed throughout the buds of control prostates with higher levels in the tips, while *c-Myc* is expressed in the bud tips (Figure 3). Expression of both genes are lost in the buds of *β-Cat;Nkx3.1:Cre* mutant prostates (n = 4), with only a few small remaining patches of *c-Myc* expression, presumably a result of epithelial cells that have not deleted β-Catenin. Interestingly, expression analysis of *Nkx3.1*, a gene important in prostate duct morphology [5], showed a dramatic loss of expression in mutant prostates (n = 5), while mutant buds still expressed *Sox9* (n = 5), another key regulator of prostate development (Figure 3) [39]. In addition, *Fgfr2* expression is downregulated in *β-Cat;Nkx3.1:Cre* mutant buds (n = 5), while *Shh* is expressed at similar levels to control prostates (n = 4) (Figure 3). This demonstrates that β-Catenin controls a discrete network of transcription factors and signalling pathways, including *Nkx3.1* and *Fgfr2*, to promote prostate bud development.

### Stabilized β-Catenin causes transdifferentiation of embryonic prostate epithelium

**Figure 4:**
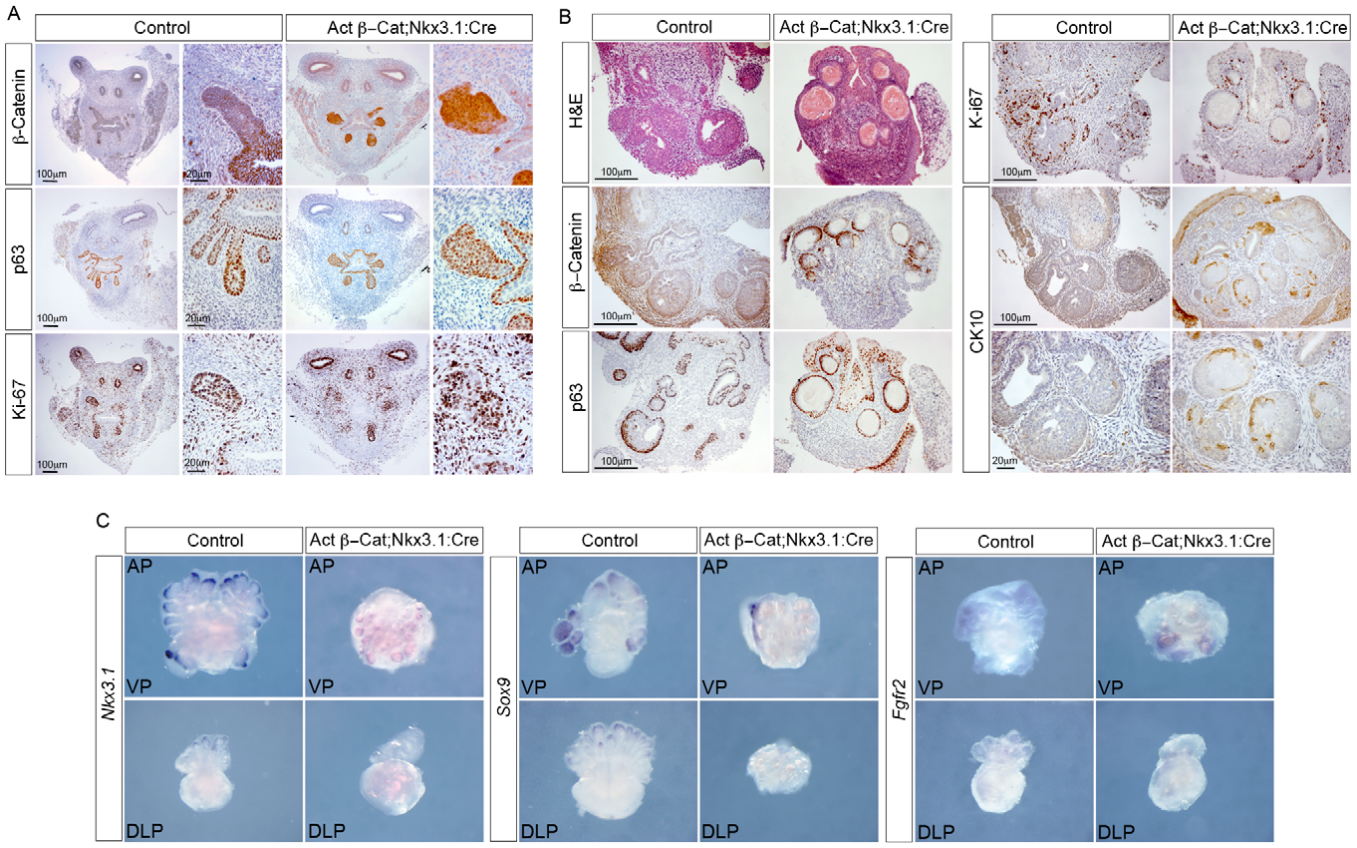
Stabilized β-Catenin transdifferentiates the developing prostate buds into squamous epithelium. (A) IHC on *Actβ-Cat;Nkx3.1:Cre* mutant and control (*Actβ-Cat*) E18.5 UGS sections with β-Catenin, p63 and Ki-67 antibodies. Adjacent panels show high magnification of buds. (B) H&E stain and IHC for β-Catenin, p63, Ki-67 and CK10 on sections of *Actβ-Cat;Nkx3.1:Cre* and control prostate organ cultures grown for 5 days. High magnification of CK10 is shown in the lower panels. (C) WISH analysis of *Actβ-Cat;Nkx3.1:Cre* mutant and control prostate organ cultures grown for 5 days shows expression of *Nkx3.1* and *Sox9* are lost in mutants, while *Fgfr2* is still present. VP is the ventral lobe, AP is the anterior lobe and DLP is the dorsal lateral lobe.

In a complementary approach to our β-Catenin deletion model, we used *Nkx3.1:Cre* to express a stabilized form of the protein (*Actβ-Cat*) in the developing mouse prostate epithelium, *Actβ-Cat;Nkx3.1:Cre* [40]. The *Actβ-Cat* allele has *loxP* sites flanking exon 3 and Cre-mediated deletion results in the loss of the GSK3β regulatory phosphorylation sites, leading to stable expression of β-Catenin. At E18.5, the epithelial buds of *Actβ-Cat;Nkx3.1:Cre* mutants are larger than control prostates (*Actβ-Cat* animals with no *Cre*) and the morphology of the bud tip is irregular (Figure 4A). The bigger *Actβ-Cat;Nkx3.1:Cre* buds express high levels of β-Catenin, contain many p63 expressing cells and have levels of proliferation comparable to control buds (*Actβ-Cat;Nkx3.1:Cre* buds 75% Ki-67 positive cells, control buds 58% Ki-67 positive cells, p = 0.12) (Figure 4A). Similar to *β-Cat;Nkx3.1:Cre* mutant pups, *Actβ-Cat;Nkx3.1:Cre* pups die at birth, so prostates were again dissected and grown in culture to assess the phenotype at later stages of development. After 5–6 days in culture, abnormal round structures developed in the place of normal prostate bud growth (Figure 4B). β-Catenin and haematoxylin and eosin (H&E) stained sections demonstrate that the epithelium of these structures has a stratified squamous morphology (Figure 4B). The normally round epithelial cells are now multi-layered and flat. Detailed analysis shows a correlation of high β-Catenin expression and the change in cell morphology, shown clearly by p63 expression (Figure S1A). Areas of squamous epithelia have lower levels of proliferation as marked by Ki-67 when compared to control prostates (Figure 4B). Furthermore, they express the squamous cell marker CK10 (Figure 4B). The abnormal structures and squamous epithelia form in *Actβ-Cat;Nkx3.1:Cre* mutants even when grown in the absence of dihydrotestosterone (DHT) (Figure S1B). *Nkx3.1* and *Sox9* expression was reduced in *Actβ-Cat;Nkx3.1:Cre* organ cultures (n = 4), while *Fgfr2* continues to be expressed in the novel structures of these mutants (n = 4) (Figure 4C). Loss of expression of these markers suggests that these cells have lost prostate identity. Overall, these data demonstrate that overexpression of β-Catenin in the developing prostate epithelia causes an androgen-independent transdifferentiation to squamous cells.

### β-Catenin is not required in the adult prostate

**Figure 5:**
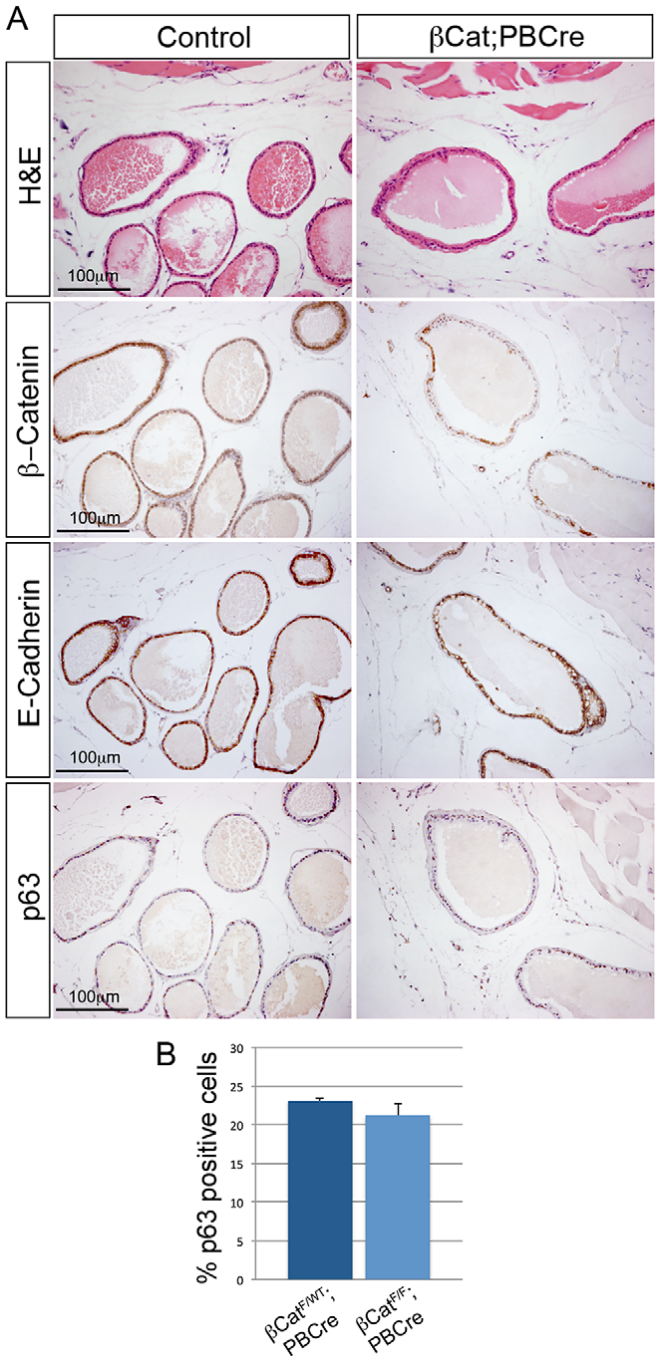
β-Catenin is not required for adult prostate homeostasis. (A) H&E stain and IHC for β-Catenin, E-Cadherin and p63 on sections of *β-Cat;PBCre* mutants and control prostates. Sections are cut through the dorsal-lateral lobe from 6 months old animals. (B) quantification of p63 expressing cells shows a similar number of basal cells in *β-Cat;PBCre* mutants as controls (p = 0.107). Error bars represent standard deviation.

To test whether β-Catenin is required for normal adult prostate homeostasis, we deleted it using the loss of function allele described (*β-Cat*) and mice with the *PBCre* transgene, which drives expression of Cre recombinase in mouse prostate epithelia from 2 weeks of age (*β-Cat;PBCre*). H&E stained sections of *β-Cat;PBCre* prostates 6 months, 9 months and 19 months of age show no observable phenotype compared to control littermates (Figure 5 and Figure S2). Analysis of β-Catenin expression in mutant prostates confirmed that it is deleted in adult prostate epithelial cells (Figure 5). In *β-Cat;PBCre* prostates E-Cadherin is correctly localized at the cell membrane suggesting the adherens junctions form (Figure 5). As our developmental studies revealed that β-Catenin regulates the number of p63 positive cells, we analysed p63 expressing basal cells in *β-Cat;PBCre* mutant and control prostates. Identical to control prostates, p63 is restricted to expression in basal cells of *β-Cat;PBCre* prostates (Figure 5). Although β-Catenin is deleted in a subpopulation of basal cell due to the mosaic expression of *PBCre*, quantification shows there are a similar number of basal cells in mutant prostates compared to controls (*β-Cat;PBCre* 23% p63 positive cells, control prostates 21% p63 positive cells, p = 0.107) (Figure 5B). In addition, *β-Cat;PBCre* prostates have similar expression of c-Myc, LEF1, AR and Probasin compared to control prostates, and mutant animals are fertile (Figure S2). These data indicate that β-Catenin has no role in maintaining normal morphology of the adult prostate.

### Pten regulated β-Catenin is not required for prostate tumorigenesis

**Figure 6:**
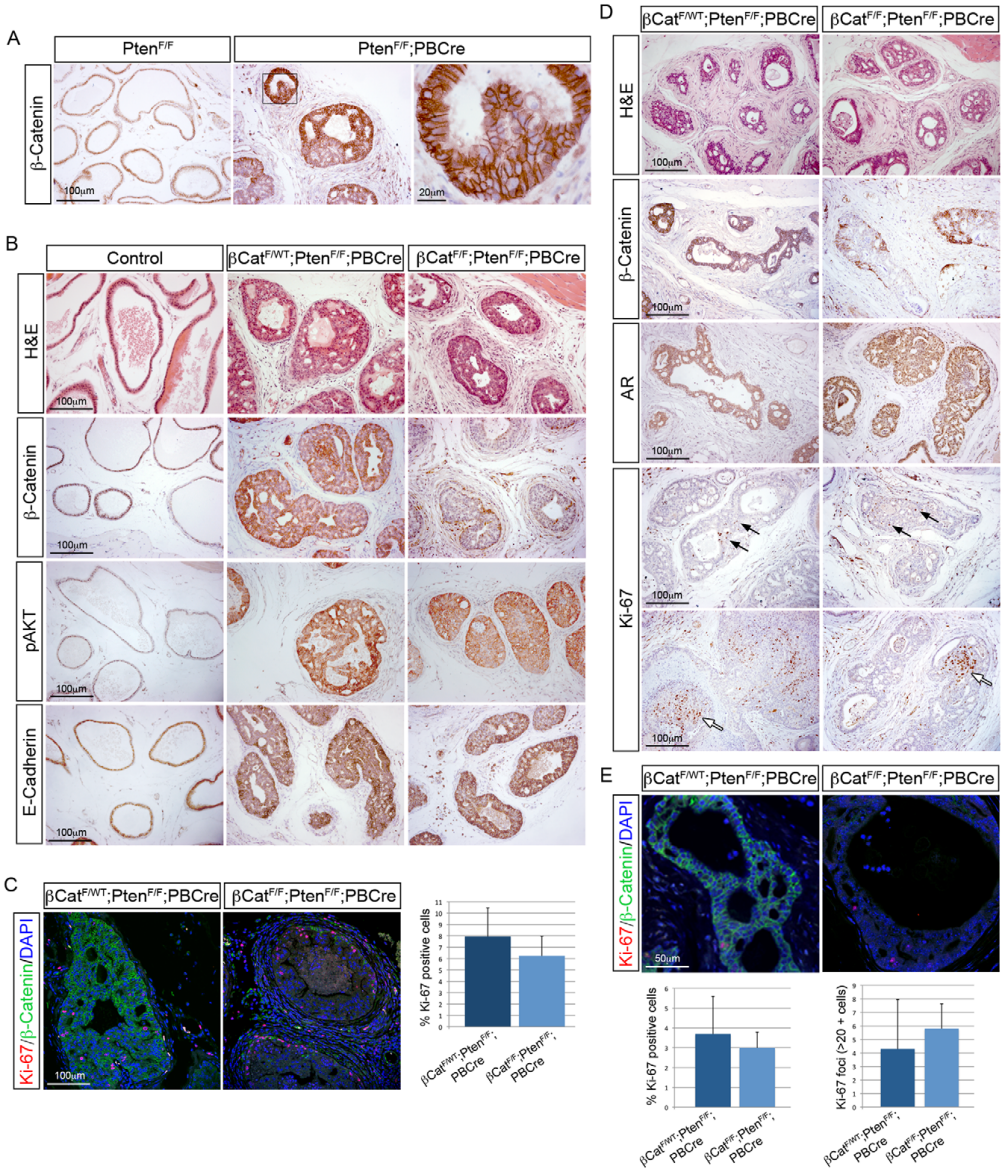
Pten regulated β-Catenin is not required for prostate tumorigenesis. (A) IHC of β-Catenin on sections of *Pten^F/F^* and *Pten^F/F^;PBCre* prostates showing upregulation of β-Catenin after *Pten* loss. (B) H&E stain and IHC for β-Catenin, pAKT and E-Cadherin on sections of 3-month-old control (no Cre), *β-Cat^F/WT^;Pten^F/F^;PBCre* and *β-Cat^F/F^;Pten^F/F^;PBCre* prostates. (C) double IHC for β-Catenin and Ki-67 on *β-Cat^F/WT^;Pten^F/F^;PBCre* and *β-Cat^F/F^;Pten^F/F^;PBCre* prostate sections. Right, quantitative analysis of Ki-67 shows no significant difference in proliferation in *β-Cat^F/F^;Pten^F/F^;PBCre* prostates compared to *β-Cat^F/WT^;Pten^F/F^;PBCre* prostates (p = 0.196). (D) H&E stain and IHC for β-Catenin, AR and Ki-67 on prostates sections of 3-month-old *β-Cat^F/WT^;Pten^F/F^;PBCre* and *β-Cat^F/F^;Pten^F/F^;PBCre* animals that were castrated for one month. Black arrows highlight proliferating cells and white arrows indicate highly proliferative foci. (E) double IHC for β-Catenin and Ki-67 on prostates sections of 3-month-old *β-Cat^F/WT^;Pten^F/F^;PBCre* and *β-Cat^F/F^;Pten^F/F^;PBCre* animals that were castrated for one month. Below left, quantitative analysis of Ki-67 shows no significant difference in proliferation in castrated *β-Cat^F/F^;Pten^F/F^;PBCre* prostates compared to *β-Cat^F/WT^;Pten^F/F^;PBCre* prostates (p = 0.695). Below right, quantitative analysis of Ki-67 foci shows no significant difference between *β-Cat^F/F^;Pten^F/F^;PBCre* and *β-Cat^F/WT^;Pten^F/F^;PBCre* prostates (p = 0.562). Error bars represent standard deviation.

To better understand the role of β-Catenin in human prostate cancer, we wanted to explore its relationship with the frequently mutated *PTEN* gene. To do this we used the well-defined Pten murine prostate cancer model [41]. In this model, *Pten* deletion in mice results in the formation of hyperplasia, prostatic intraepithelial neoplasia (PIN) and carcinoma. *Pten* null prostates were generated using a *loxP* containing *Pten* allele and the *PBCre* transgene (*Pten^F/F^;PBCre*). We first analysed the expression of β-Catenin protein to determine if β-Catenin levels are altered in these animals, as studies in prostate cancer cell lines have shown that *Pten* loss leads to β-Catenin accumulation [33], [34]. Immunohistochemistry shows that *Pten^F/F^;PBCre* prostates have a dramatic increase in the level of β-Catenin compared to controls, which is confirmed by Western blot (Figure 6A and Figure S3A). In addition, in *Pten* null prostate tumours there is an increase in the active non-phosphorylated stabilized form of β-Catenin, which is thought to be involved in the transcription of target genes, although we could not detect an induction of LEF1 (Figure S3B). To test whether this increase in β-Catenin expression has a role in *Pten* loss-driven prostate cancer, we deleted *Ctnnb1* in the *Pten* null model using the *loxP* alleles and the *PBCre* transgene described. Detailed histopathological analysis of *Pten* mutant (*Pten^F/F^;PBCre* and *β-Cat^F/WT^;Pten^F/F^;PBCre*), *Pten;Ctnnb1* double mutant (*β-Cat^F/F^;Pten^F/F^;PBCre*) and control animals (*β-Cat^F/F^;Pten^F/F^*) aged 3 months demonstrates a similar progression to PIN in *Pten* null mutants and in *β-Cat^F/F^;Pten^F/F^;PBCre* mutants (Figure 6B). pAKT levels remain high and E-Cadherin expression remain unaltered in both *β-Cat^F/WT^;Pten^F/F^;PBCre* mutant prostates and in *β-Cat^F/F^;Pten^F/F^;PBCre* mutant prostates (Figure 6B). Consistent with this, the percentage of Ki-67 positive cells in *Pten* null prostates, which is comparable to previous reports, is not significantly different to prostates that have lost *Pten* and *Ctnnb1* (Figure 6C) [41], [42]. This shows there is no difference in the progression to PIN in prostates that have lost *Pten* and those that have lost both *Pten* and *Ctnnb1*, suggesting that β-Catenin does not play a role in *Pten* loss-driven prostate cancer.

Castration of mice with *Pten* null prostates results in regression of PIN, but cells continue to proliferate [41]. As β-Catenin has been implicated in the progression of CRPC, we investigated if β-Catenin has a role in the continued growth of *Pten* deficient prostates. To do this, we surgically castrated 3 month old *β-Cat^F/WT^;Pten^F/F^;PBCre* and *β-Cat^F/F^;Pten^F/F^;PBCre* mice and analysed the prostates 1 month later. H&E and β-Catenin stained sections demonstrate that both *Pten* null and *β-Cat^F/F^;Pten^F/F^;PBCre* mutant prostates regress (Figure 6D). Castration of animals with *Pten* null prostates resulted in AR protein being retained in the cytoplasm and a more diffuse expression pattern [41]. This response to androgen withdrawal is also seen in *β-Cat^F/F^;Pten^F/F^;PBCre* mutant prostates and suggests a similar effect on AR in both models. Analysis of Ki-67 expression in *β-Cat^F/WT^;Pten^F/F^;PBCre* and *β-Cat^F/F^;Pten^F/F^;PB4Cre* prostates shows that cellular proliferation continues after castration (Figure 6D). In addition, there were often foci of many Ki-67 positive cells in animals of both genotypes (more than 20 positive cells) (Figure 6D). In *β-Cat^F/F^;Pten^F/F^;PBCre* mutant prostates, Ki-67 foci were present in areas of epithelia that has lost β-Catenin as well as those that had retained expression. Quantification of Ki-67 shows there is no significant difference in total proliferation or in Ki-67 foci between *Pten* null prostates and *β-Cat^F/F^;Pten^F/F^;PBCre* mutant prostates after castration (Figure 6E). Taken together, this work demonstrates that *Pten* loss results in increased levels of β-Catenin in the prostate, but this is not required for tumour growth in intact or castrated animals.

### Stabilized β-Catenin cooperates with *Pten* loss to drive prostate cancer progression

**Figure 7:**
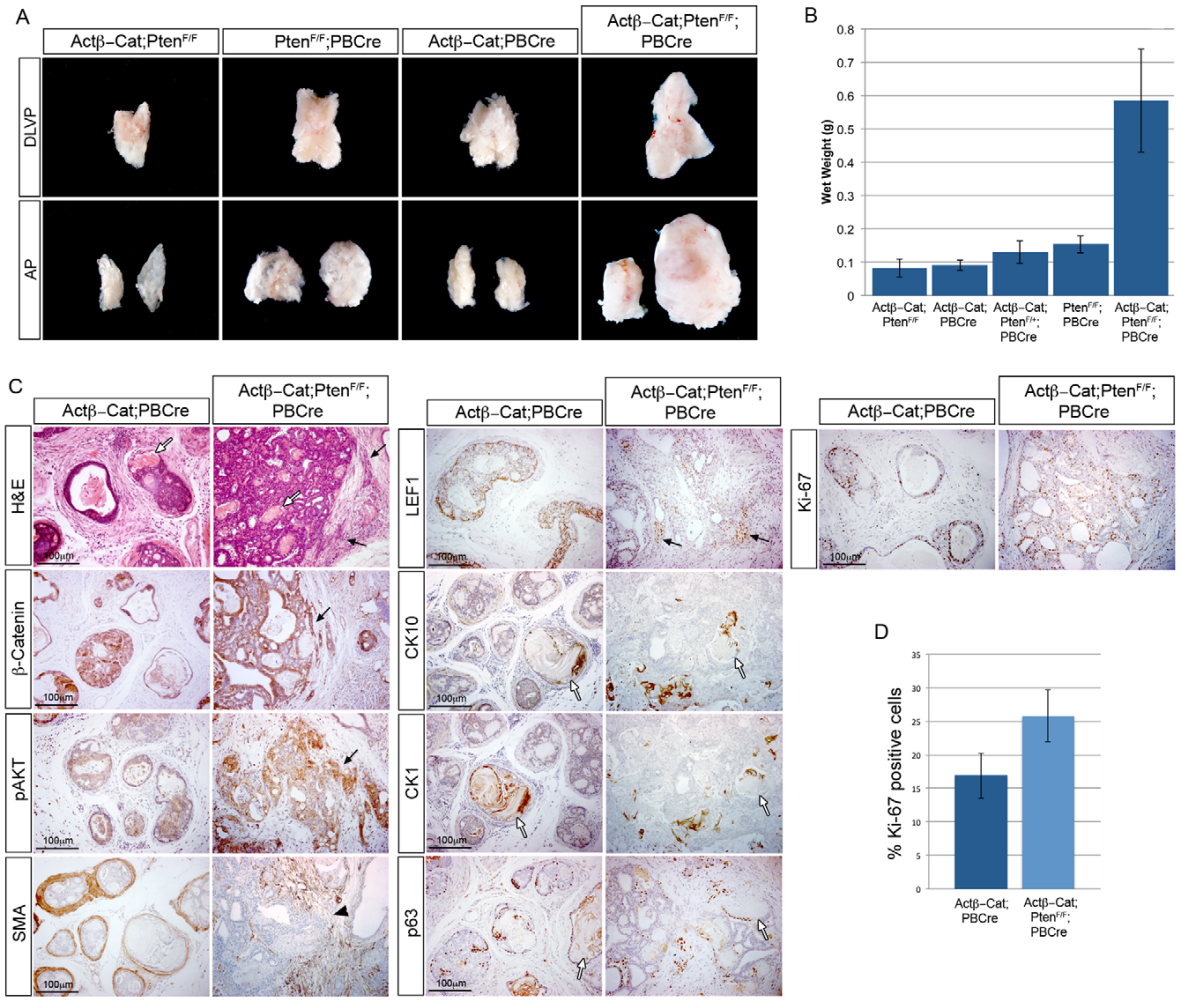
Stabilized β-Catenin cooperates with *Pten* loss to drive prostate cancer progression. (A) brightfield images of mouse prostates with epithelial *Pten* deletion and stabilized β-Catenin, as indicated. The dorsal-lateral-ventral lobes (DLVP) and anterior lobes (AP) from individual animals of each genotype are shown. (B) wet weights of prostates with epithelial *Pten* deletion and stabilized β-Catenin. (C) H&E stain and IHC for β-Catenin, pAKT, smooth muscle actin (SMA), LEF1, CK10, CK1, p63 and Ki-67 on sections of 3-month-old *Actβ-Cat;PBCre* and *Actβ-Cat;Pten^F/F^;PBCre* prostates. (D) quantitative analysis of Ki-67 shows a significant increase in proliferation in *Actβ-Cat;Pten^F/F^;PBCre* prostates compared to *Actβ-Cat;PBCre* (p = 0.039). Black arrows indicate epithelial cells invading into the stroma. White arrows indicate areas of squamous metaplasia. Black arrowhead indicates loss of SMA. Error bars represent standard deviation.

*Pten* null animals form high-grade PIN but do not readily progress to invasive carcinoma [42]. Although we found the higher β-Catenin levels after *Pten* deletion did not have a function in prostate cancer initiation, we wanted to test whether increasing levels of β-Catenin further would affect progression. To do this, we crossed the *Pten loxP* (*Pten^F/F^*) animals with mice with the stabilized form of β-Catenin (*Actβ-Cat*) and the *PBCre* transgene. This allowed the comparison of prostates that are *Pten* null (*Pten^F/F^;PBCre*), have stabilized β-Catenin (*Actβ-Cat;PBCre*), *Pten* null in combination with stabilized β-Catenin (*Actβ-Cat;Pten^F/F^;PBCre*) and controls (*Actβ-Cat;Pten^F/F^*). *Actβ-Cat;Pten^F/F^;PBCre* prostates have higher levels of active non-phosphorylated β-Catenin than *Pten* null prostates and show a clear induction of LEF1, indicating these tumours have an increase in β-Catenin mediated transcription (Figure S3B). At 2 months of age, *Actβ-Cat;Pten^F/F^;PBCre* prostates display high-grade PIN and focally invasive epithelial cells in the stroma (Figure S4A). By 3 months of age, *Actβ-Cat;Pten^F/F^;PBCre* prostates are larger and weigh significantly more than *Pten^F/F^;PB4Cre* or *Actβ-Cat;PBCre* prostates (Figure 7A and 7B). H&E staining shows that *Actβ-Cat;Pten^F/F^;PBCre* prostates have large areas of high-grade PIN and invasive adenocarcinoma with extensive infiltrative growths of atypical epithelial cells invading into the surrounding fibromuscular stroma and connective tissue, which are not present in *Pten^F/F^;PBCre* or *Actβ-Cat;PBCre* prostates (Figure 6B and Figure 7C). These invasive areas express high levels of β-Catenin, LEF1 and pAKT, indicating that these pathways cooperate to promote the invasive phenotype (Figure 7C and Figure S4B). In double mutant prostates, smooth-muscle actin staining is broken-up and irregular, confirming that epithelial cells have spread into the stroma (Figure 7C). In addition, *Actβ-Cat;Pten^F/F^;PBCre* lesions are highly proliferative and have a significant increase in the number of proliferating cells compared to *Actβ-Cat;PBCre* prostates (*Actβ-Cat;PBCre* 17% Ki-67 cells, *Actβ-Cat;Pten^F/F^;PBCre* 26% Ki-67 cells, p = 0.039) (Fig. 7C and 7D). Comparable with embryonic prostates expressing stabilized β-Catenin, *Actβ-Cat;PBCre* and *Actβ-Cat;Pten^F/F^;PBCre* prostates display areas of squamous differentiation and keratinization that express the squamous cell markers CK10 and CK1 and that frequently associate with many p63 positive cells (Figure 7C and Figure S4B). These data demonstrate that β-Catenin can interact with *Pten* loss to form highly invasive prostate cancer and squamous metaplasia.

## Discussion

The role of β-Catenin in prostate development is not known and its function in prostate cancer is not clearly defined. We have undertaken an in depth genetic study in the embryonic and adult mouse to identify the role β-Catenin plays in these processes. Our developmental genetic studies demonstrate that epithelial β-Catenin is essential for early growth of the prostatic buds but that high levels of β-Catenin lead to the formation of squamous epithelia. Deletion of *Ctnnb1* in the adult prostate has no effect on normal prostate homeostasis or a *Pten* model of prostate cancer. However, the functional significance of β-Catenin in prostate cancer is highlighted as increased levels can cooperate with *Pten* loss to drive progression to invasive carcinoma together with squamous transdifferentiation. The ability of β-Catenin to differentiate prostate epithelia into squamous cells in the embryo and adult suggests that this protein is playing a similar role in both developmental stages. These studies further highlight the use of developmental systems to inform on the function of genes and pathways in the adult.

Our studies show that during prostate development β-Catenin controls the proliferation of cells at the tip of the epithelial buds and regulates the number of p63 positive cells in this region. This lack of proliferation leads to a defect in prostatic bud growth and branching in mutant animals, consistent with other studies that have shown that the tips of the buds have high numbers of proliferating cells and are important in the formation of branches [43]. Our data reveal that β-Catenin regulates the expression of LEF1 and *c-Myc* at the tip of the developing prostate bud. c-Myc is a well-known regulator of proliferation and it is therefore likely that β-Catenin controls cell number in prostate buds through this gene [44], [45]. We were unable to determine if β-Catenin is required for prostate induction and bud initiation as the *Nkx3.1:Cre* mouse line is first expressed after this has begun at E17.5. A recent study using an inducible system to delete β-Catenin prior to prostate formation has demonstrated that this protein is required for bud initiation [46]. Together, these data demonstrate that β-Catenin is required for multiple steps of prostate development, including prostatic epithelial progenitor cell proliferation at the tip of the buds and for ductal branching.

Prostate development is regulated by a set of transcription factors and signalling networks that are poorly defined. Two of the earliest markers of prostate identity are the transcription factors *Nkx3.1* and *Sox9*. These, together with FGF signalling, through Fgfr2, and the Hedgehog signalling pathway, through Shh, are required for normal prostate development [6]. Our work has uncovered a novel divergence in the pathways that control prostate development, with β-Catenin regulating the expression of *Nkx3.1* and *Fgfr2* but having no effect on *Sox9* and *Shh* expression. This suggests a complex gene network during prostate development with different pathway branches regulating prostate identity in parallel, and β-Catenin regulating *Nkx3.1* and *Fgfr2* in one of these branches. FGF signalling has been implicated in the early formation of prostate buds and β-Catenin could be acting through this pathway [47]. However, the prostate phenotype in mice where *Fgfr2* was deleted using the *Nkx3.1:Cre* strain was not the same as in *β-Cat;Nkx3.1:Cre* mutants, suggesting that Fgfr2 is not the only factor responsible for the defects in growth and branching in the latter animals [47].

Our studies indicate that β-Catenin acts through the canonical WNT pathway to regulate prostate epithelial cell proliferation, as we observe the loss of expression of classical downstream genes of this pathway after *Ctnnb1* deletion, including LEF1, *c-Myc* and *Axin2*. In addition, several members of the WNT family have been shown to be expressed in the epithelia and mesenchyme of the developing prostate [48]. Our results are consistent with published studies where addition of WNT ligand to rat prostate cultures was shown to increase the number of p63 positive cells, while the WNT signalling inhibitor Dkk had the opposite effect, and led to disrupted prostatic branching [49]. From our gene deletion experiments, it may have been expected that expressing stabilized β-Catenin during embryogenesis would result in an expansion of the prostatic ducts. Although we observe this at E18.5, at later stages the epithelium becomes squamous-like. Our studies suggest that WNT signalling needs to be tightly regulated during prostate development and negative regulators of the pathway, such as Dkk1, may be required to control the level of β-Catenin and prevent prostate epithelia transdifferentiating to a squamous phenotype.

Our data demonstrate that β-Catenin regulates the number of p63 positive progenitor cells in the developing prostate. Interestingly, WNT/β-Catenin signalling has been shown to control self-renewal of stem/progenitor cells of primary adult prostate epithelial cells and prostate cancer cell lines in prostate sphere assays [50], [51]. These studies have shown that Bmi-1 is necessary for β-Catenin self-renewal activity. Bmi-1 can induce the self-renewal activity of prostate spheres and results in the expansion of p63 positive cells. In this assay, the p63 expressing cells have a higher capacity for self-renewal, indicating they contain adult prostatic progenitor cells [52]. Together, these data suggest that pathways that regulate progenitor cells during embryonic prostate development also control stem/progenitor cells in the adult prostate.

We have shown that during prostate development β-Catenin overexpression results in squamous epithelia differentiation, even in the absence of androgens. Similarly, squamous cells form when stabilized β-Catenin is expressed in the adult prostate, even after *Pten* loss. This suggests that an intrinsic property of high levels of β-Catenin in prostate epithelia is to drive squamous differentiation. Squamous metaplasia is also seen after exogenous administration of estrogen to male mice. Estrogen has been shown to act through estrogen receptor alpha (ERα) in the epithelium to promote proliferation of p63 expressing basal cells and the formation of squamous metaplasia [53], [54], [55]. Interestingly, we found an increase in ERα expression in *Actβ-Cat;PBCre* prostates, however, this was not the case for *Actβ-Cat;Pten^F/F^;PBCre* mice, suggesting that high ERα is not a requisite for squamous cell formation driven by high levels of β-Catenin (data not shown). Ectopic high levels of p63 expression were able to induce transdifferentiation and squamous metaplasia of mouse lung epithelium [56]. In addition, high p63 levels have been associated with squamous cell carcinoma and β-Catenin has been shown to regulate this protein in this disease [57], [58]. Therefore our studies suggest that stabilized β-Catenin acts to induce high levels of p63, which drives the transdifferentiation process towards a squamous fate.

β-Catenin has been implicated in CRPC and has been shown to act as an AR co-factor in low androgen conditions [17], [18], [19], [20], [21], [22], [24]. *Pten* mutant mice show resistant properties with proliferation of epithelial cells in the prostates of castrated animals [41]. Our data demonstrate that although β-Catenin is upregulated after *Pten* loss, it is not essential in neoplastic growth of *Pten* null cancer after androgen withdrawal. This data suggests that, in this CRPC model, β-Catenin does not act as a co-factor with AR or in an AR-independent manner to regulate neoplastic proliferation. The increase in β-Catenin expression we observe after *Pten* deletion is predominantly localized at the membrane and in the cytoplasm, with little transcriptionally active protein accumulating. This low level of active β-Catenin may not induce transcription of targets, such as LEF1, and have no affect on *Pten*-loss driven PIN. This is in contrast to our *Actβ-Cat;Pten^F/F^;PBCre* model that has high levels of nuclear β-Catenin, increased levels of LEF1 expression and progression to an aggressive phenotype. Alternatively, the growth of *Pten* null prostate lesions deficient in β-Catenin may be due to compensation by the multiple downstream effectors of the PI3K/Akt pathway that are affected by *Pten* loss [32].

Our study, and work by others, has shown that activating β-Catenin through a stabilized form of the protein in the prostate leads to PIN and squamous metaplasia, with lesions that are highly proliferative but not invasive [27]. Interestingly, our data demonstrates that increased β-Catenin levels are able to drive *Pten* deficient prostate epithelia, a frequent genetic lesion in human prostate cancer, to invasive carcinoma suggesting that these pathways could interact during human prostate tumorigenesis. Consistent with this, recent exome sequencing has shown that members of the WNT pathway, including APC, are frequently mutated in aggressive prostate tumours, and these cancers are enriched for mutations in a PTEN interacting network [59]. β-Catenin has also been shown to cooperate with mutant *K-ras* and SV40 large T-antigen to drive tumour progression to invasive carcinoma [28], [29]. Mice expressing active β-Catenin and mutant *K-ras* in prostate epithelial cells have a loss of p63 expressing cells and do not display squamous metaplasia, consistent with our hypothesis that p63 expression is involved in transdifferentiation to squamous cells [29]. The combination of active β-Catenin and SV40 large T-antigen results in epithelial cell invasion into the stroma [28]. Similar to our model, nuclear β-Catenin and expression of downstream targets were found in the epithelial cells within the stroma suggesting that this pathway can act synergistically with multiple signalling pathways to promote the formation of invasive aggressive prostate cancer.

The ability of β-Catenin to cooperate with other oncogenic events such as *Pten* deletion, mutant *K-ras* and SV40 large T-antigen to drive tumour progression suggests that it may be a general drug target in prostate cancer [28], [29]. Changes in β-Catenin expression are typically a late event in human prostate cancer and detailed sequencing studies have shown that mutations in the WNT pathway occur in late stage lethal CRPC [59], [60]. This suggests that inhibitors of this pathway, such as tankyrase inhibitors, could be used in the clinic to treat patients with these mutations [61]. Squamous metaplasia was also observed in these mice, which is rarely seen in human prostate cancer. Our data therefore suggests that other events need to take place to allow β-Catenin to drive disease progression without squamous formation. In the model we used, *Pten* and *Actβ-Cat* undergo Cre-mediated recombination at the same time within luminal cells, although some basal cells are also targeted. Therefore genetic lesions may need to occur in a step-wise fashion in patients so that β-Catenin expression is altered at a later stage in tumour formation. Alternatively, β-Catenin may need to interact with other genetic lesions or be upregulated in a different cell type to give rise to tumours that do not exhibit squamous differentiation. These studies provide novel information on the necessary steps in tumour development that are required for progression to the invasive phenotype seen in human patients with lethal prostate cancer.

## Materials and Methods

### Mouse strains

Mice containing the *Ctnnb1* conditional allele (*β-Cat*) have been described previously [35]. Mice containing the stabilized *Ctnnb1* conditional allele (*Actβ-Cat*) were kindly provided by M.M. Taketo [40]. The *ARR2PBCre* transgenic mice, *PBCre*, have been described previously [62]. The *Nkx3.1:Cre* allele was created by introducing the *Cre* gene by homologous recombination into the *Nkx3.1* locus and was kindly provided by Michael Shen. Mice with the conditional allele of *Pten* were obtained from The Jackson Laboratory [63]. These animals were bred on a mixed genetic background. We define midday of the day of plug as E0.5 and most pregnant females in this study gave birth on E19. All procedures were in accordance with UK Home Office legislation.

### Organ culture system

E18.5 urogenital sinuses were dissected from male embryos and grown on Biopore membranes (MilliPore) with culture media (DMEM/F-12 HAM 1/1 mixture) supplemented with ITS+1 (Sigma-Aldrich I-2521) at 20 µl/ml, gentamicin (0.05 mg/ml), benzylpenicillin sodium (0.12 mg/ml), streptomycin sulphate (0.2 mg/ml), ampicillin (0.1 mg/ml) and 10^−8^ M dihydrotestosterone.

### Whole-mount *in situ* hybridisation (WISH)

WISH was carried out on an in situ processor (Intavis In Situ Pro) according to standard protocols [64]. Probes for *Sox9* and *Nkx3.1* have been described previously [5], [65]. The *Axin2*, *c-Myc*, *Fgfr2* and *Shh* DIG containing probes were synthesized from PCR fragments containing T7 RNA polymerase promoter sequences as described previously [64]. These fragments were derived from the amplification of mouse cDNA or genomic DNA using the following primers:

Axin2: 5′ AGGATGCTGAAGGCTCAAAGC 3′ and

5′ GTAATACGACTCACTATAGGGACTCTGGATGGTCCCCAAAG 3′

c-Myc: 5′ AAGCTGGTCTCGGAGAAGCTGG 3′ and

5′ TAATACGACTCACTATAGGGAGGTTCAGGGATCTGGTCACG 3′

Fgfr2: 5′ CGCAGGATGGACCTCTCTAC 3′ and

5′ GTAATACGACTCACTATAGGGCGACCAACTGCTTGAATGTG 3′;

Shh: 5′ GACAGCTCACAAGTCCTCAG 3′ and

5′ GTAATACGACTCACTATAGGGACGTAAGTCCTTCACCA 3′.

For each WISH probe, at least four embryos of each genotype were processed.

### Mouse prostate histology

Histological phenotype of samples was assessed on H&E stained sections. Serial sections were then stained for immunohistochemical analysis. Histological assessment was based on published guidelines and assisted by the pathologists R. Cardiff, D. Berney and G. Stamp [66], [67].

### Immunohistochemistry and Western blot

Antibody stains were performed on paraffin sections. Tissues were fixed overnight in 4% paraformaldehyde (PFA), dehydrated by washing through an ethanol gradient series, washed in histoclear and embedded in wax. 4 µm sections were cut, treated with histoclear and rehydrated through an ethanol gradient series. Antigen retrieval was obtained by boiling the sections in citrate buffer (0.1 M sodium citrate pH6 and 0.05% Tween) and sections were treated with 3% H_2_O_2_ to block endogenous peroxidase activity. Sections were incubated in PBS with 10% sheep serum and then incubated with primary and secondary antibodies in PBS with 1% sheep serum. Sections were counterstained with hematoxylin. The following antibodies were used for immunohistochemistry; β-Catenin (BD Biosciences, 1∶100 fluorescence, 1∶800 DAB), AR (PG-21, Upstate, 1∶250 DAB), p63 (4A4, Santa Cruz Biotechnology, 1∶50 fluorescence, 1∶200 DAB), CK10 (PRB-159P-100, Covance, 1∶1000 DAB), CK1 (PRB-165P-100, Covance, 1∶1000 DAB), LEF1 (2230, Cell Signalling Technology, 1∶100 fluorescence, 1∶1000 DAB), pAKT (Ser473) (9271, Cell Signalling Technology, 1∶100 DAB), Ki-67 (clone TEC-3, Dako, 1∶20 fluorescence, 1∶200 DAB), smooth-muscle actin (clone 1A4, Sigma, 1∶5000 DAB), E-Cadherin (BD Transduction Laboratories, 1∶250 DAB). For DAB chromogen staining the ABC vector kit was used with biotinlyated secondary antibodies (Vector Laboratories) according to manufacturer's instructions and the DAB substrate (Dako). Secondary fluorescent antibodies were obtained from Molecular Probes and were used at a 1∶500 dilution. Fluorescent images were visualized and collected on a Leica TCS-SP2 confocal microscope. For all antibody stains, sections were stained from at least four animals of each genotype. Western blotting was performed using standard protocols, and the following antibodies were used; β-Catenin 1∶500; active non-phosphorylated β-Catenin 1∶500 (Millipore), LEF1 1∶1000, HSC70 1∶15,000 (Santa Cruz Biotechnology), β-Actin 1∶500 (Santa Cruz Biotechnology).

### Quantification of proliferation and basal cells

To quantify proliferating cells, Ki-67 and β-Catenin double-labelled fluorescent immunohistochemistry was performed on sections and stained with nuclear DAPI. For developmental and adult studies prostatic ducts were identified using DAPI. For control ducts and ducts expressing stabilized β-Catenin Ki-67 positive cells were counted in the β-Catenin positive epithelia. For loss-of-function β-Catenin mutant buds Ki-67 positive cells were counted in the β-Catenin negative prostate epithelia. To quantify basal cells, p63 expressing cells were counted and shown as a percentage of the total epithelial cells. For developmental studies, cells were counted from a section of five embryos of each genotype. For adult studies, cells from at least 4 high power fields were counted per animal, which totalled more than 900 cells per animal. At least three animals of each genotype were analysed. All values are significant with p<0.05 using Student t-test unless otherwise stated.

### β-Galactosidase staining

Tissues were dissected and fixed in PBS with 2% PFA and 0.1% glutaraldehyde for 30 min at 37°C. After washing in PBS, tissues were stained at 37°C in staining buffer (1 mg/ml X-gal, 2 mM MgCl2, 5 mM K3Fe(CN)6, 5 mM K4Fe(CN)6 and 0.02% NP-40 in PBS) in the dark.

## Supporting Information

Figure S1 Embryonic squamous formation of prostate epithelium by stabilized β-Catenin. (A) high magnification of β-Catenin and p63 IHC on sections of *Actβ-Cat;Nkx3.1Cre* mutant and control (*Actβ-Cat*) prostate organ cultures grown for 3 days. Arrows indicate area of high β-Catenin that has become squamous stratified epithelium. (B) H&E and IHC for β-Catenin on sections of *Actβ-Cat;Nkx3.1Cre* prostate organ cultures grown for 5 days with no DHT.
(TIF)

Figure S2 β-Catenin is not required for adult prostate homeostasis. H&E stain and IHC for AR, Probasin, c-Myc and LEF1 on sections of *β-Cat;PBCre* mutant and control prostates. Sections cut through the dorsal-lateral lobe.
(TIF)

Figure S3 Western blot analysis of β-Catenin and LEF1 in adult mouse prostate tissue. (A) β-Catenin levels increase in *Pten* null (*Pten^F/F^;PBCre*) prostates compared to controls. (B) undetectable levels of active non-phosphorylated β-Catenin are present in control prostates, while in *Pten^F/F^;PBCre* prostates there is an accumulation of wild type endogenous active non-phosphorylated β-Catenin, as indicated. *Actβ-Cat;Pten^F/F^;PBCre* prostates have very high levels of the smaller exon 3 deleted mutant active β-Catenin, as indicated. The anti-active β-Catenin antibody also detects a smaller unknown band in control and *Pten^F/F^;PBCre* samples. LEF1 is not detected in control or *Pten^F/F^;PBCre* prostates and is upregulated *Actβ-Cat;Pten^F/F^;PBCre* prostates.
(TIF)

Figure S4 Stabilized β-Catenin and *Pten* homozygous loss prostate cancer. (A) H&E stain on sections of 2-month-old *Actβ-Cat;Pten^F/F^;PBCre* prostates. Left panel shows areas of adenocarcinoma and squamous metaplasia. Right panel shows epithelial cells invading into the surrounding stroma. (B) H&E stain and IHC for β-Catenin and p63 on sections of 3-month-old *Actβ-Cat;Pten^F/F^;PBCre* prostates showing detail of adenocarcinoma and squamous metaplasia. Black arrows indicate mitotic figures. White arrows indicate cords of β-Catenin positive epithelial cells that have invaded the stroma. Arrowheads indicate squamous metaplasia.
(TIF)
