# Supplementary figures and images for: β-Catenin Is Required for Prostate Development and Cooperates with Pten Loss to Drive Invasive Carcinoma
Source: PLoS Genet. 2013 Jan 3;9(1):e1003180. doi: 10.1371/journal.pgen.1003180 (PMC3536663; doi:10.1371/journal.pgen.1003180)

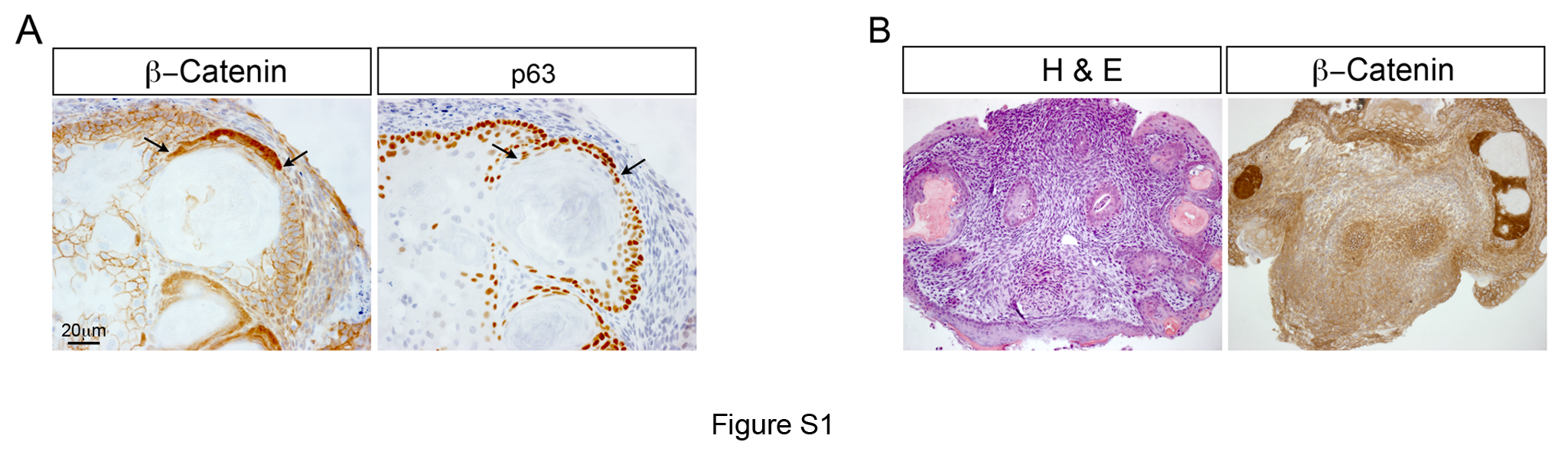

Supplement: Figure S1 — Embryonic squamous formation of prostate epithelium by stabilized β-Catenin. (A) high magnification of β-Catenin and p63 IHC on sections of Actβ-Cat;Nkx3.1Cre mutant and control (Actβ-Cat) prostate organ cultures grown for 3 days. Arrows indicate area of high β-Catenin that has become squamous stratified epithelium. (B) H&E and IHC for β-Catenin on sections of Actβ-Cat;Nkx3.1Cre prostate organ cultures grown for 5 days with no DHT. (TIF) [file pgen.1003180.s001.tif]

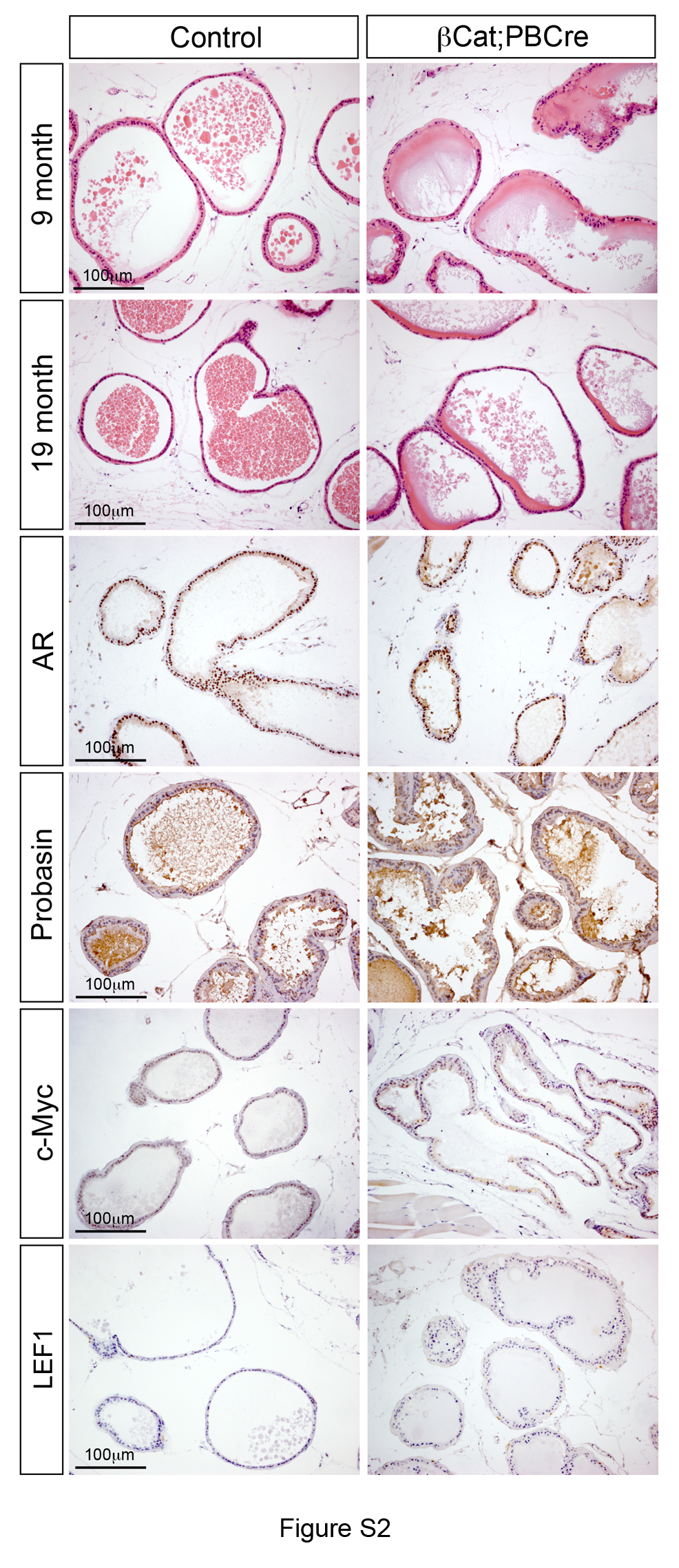

Supplement: Figure S2 — β-Catenin is not required for adult prostate homeostasis. H&E stain and IHC for AR, Probasin, c-Myc and LEF1 on sections of β-Cat;PBCre mutant and control prostates. Sections cut through the dorsal-lateral lobe. (TIF) [file pgen.1003180.s002.tif]

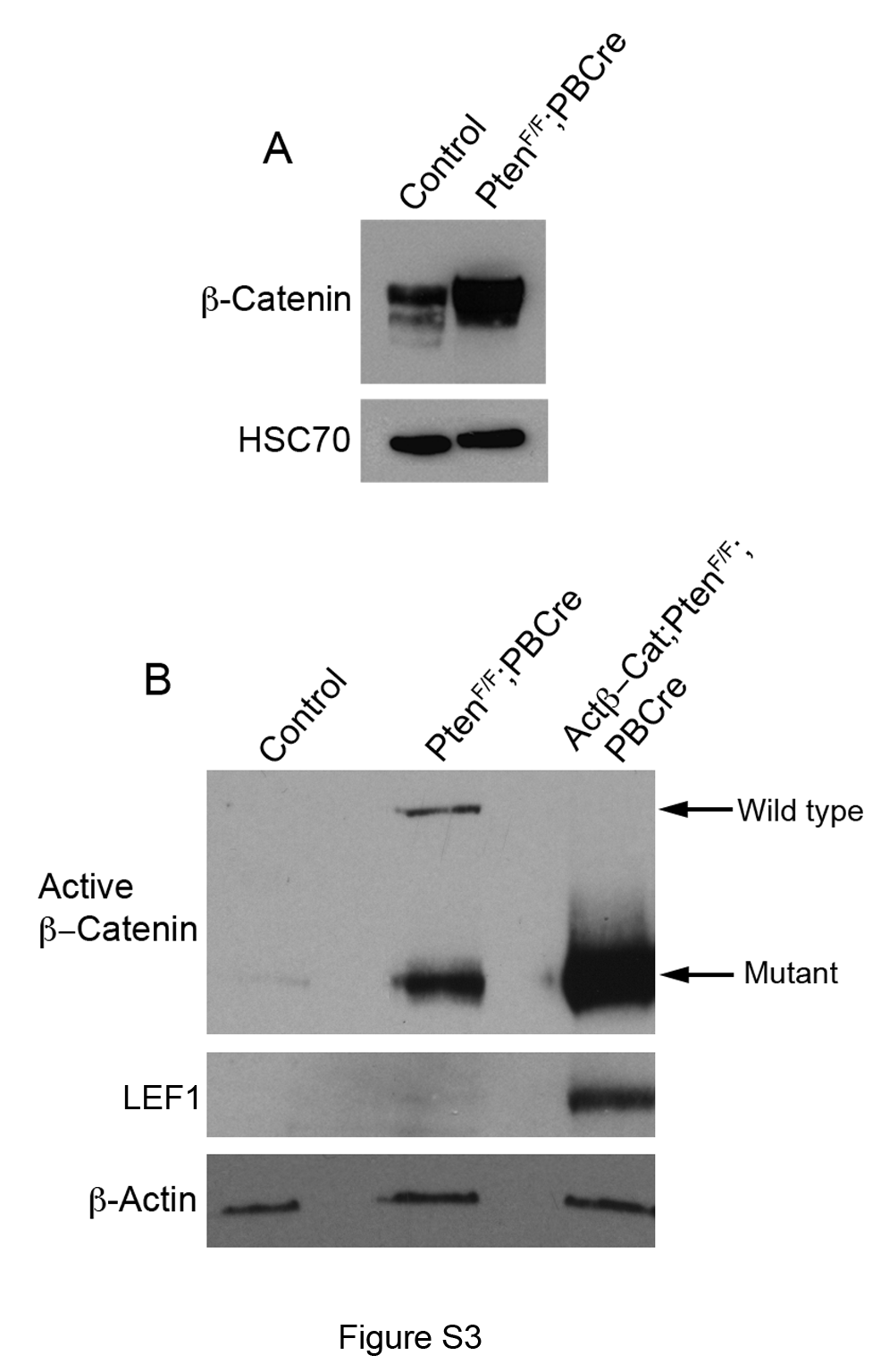

Supplement: Figure S3 — Western blot analysis of β-Catenin and LEF1 in adult mouse prostate tissue. (A) β-Catenin levels increase in Pten null (PtenF/F;PBCre) prostates compared to controls. (B) undetectable levels of active non-phosphorylated β-Catenin are present in control prostates, while in PtenF/F;PBCre prostates there is an accumulation of wild type endogenous active non-phosphorylated β-Catenin, as indicated. Actβ-Cat;PtenF/F;PBCre prostates have very high levels of the smaller exon 3 deleted mutant active β-Catenin, as indicated. The anti-active β-Catenin antibody also detects a smaller unknown band in control and PtenF/F;PBCre samples. LEF1 is not detected in control or PtenF/F;PBCre prostates and is upregulated Actβ-Cat;PtenF/F;PBCre prostates. (TIF) [file pgen.1003180.s003.tif]

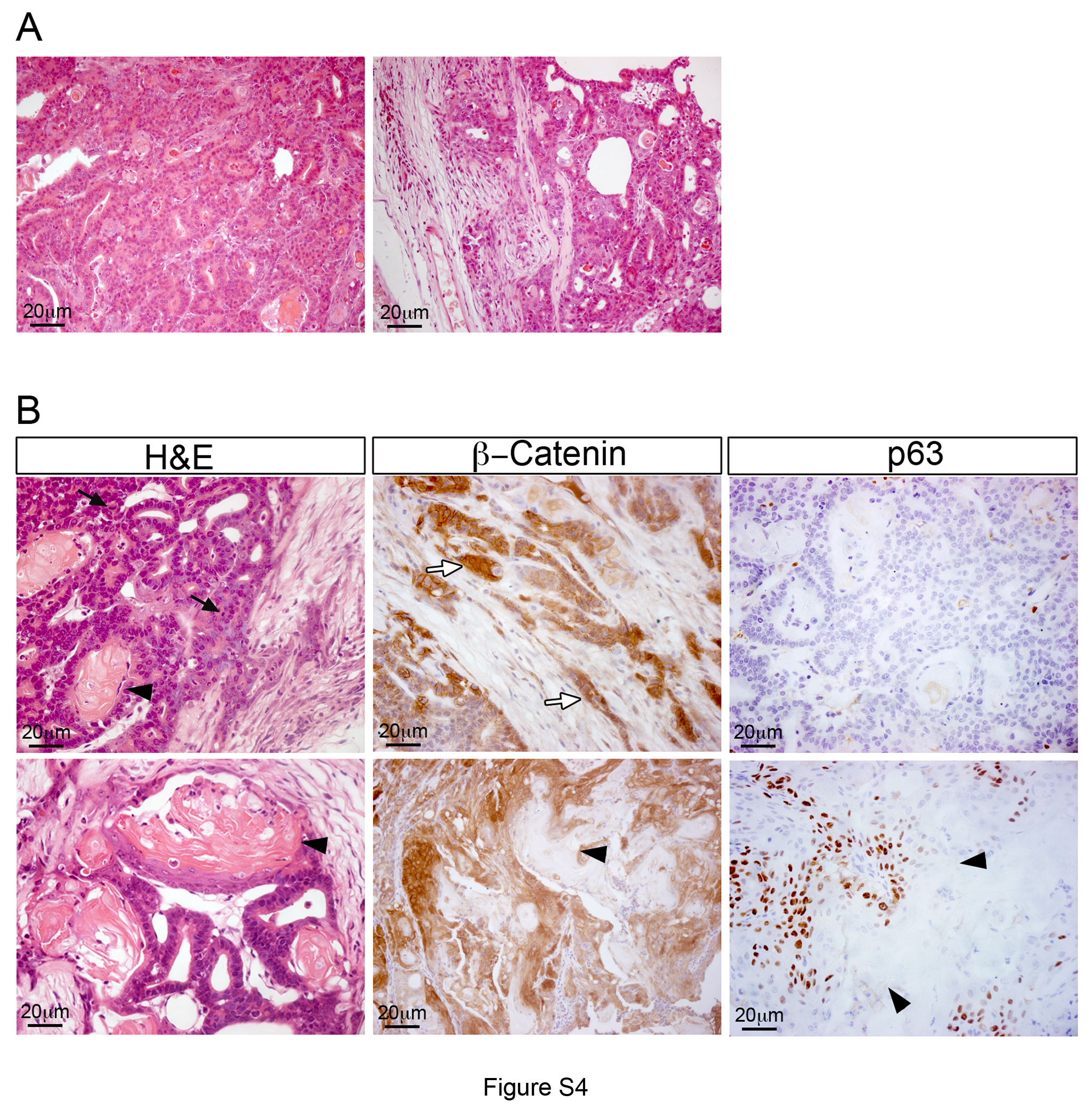

Supplement: Figure S4 — Stabilized β-Catenin and Pten homozygous loss prostate cancer. (A) H&E stain on sections of 2-month-old Actβ-Cat;PtenF/F;PBCre prostates. Left panel shows areas of adenocarcinoma and squamous metaplasia. Right panel shows epithelial cells invading into the surrounding stroma. (B) H&E stain and IHC for β-Catenin and p63 on sections of 3-month-old Actβ-Cat;PtenF/F;PBCre prostates showing detail of adenocarcinoma and squamous metaplasia. Black arrows indicate mitotic figures. White arrows indicate cords of β-Catenin positive epithelial cells that have invaded the stroma. Arrowheads indicate squamous metaplasia. (TIF) [file pgen.1003180.s004.tif]
